# Surgery

**Published:** 1880-06

**Authors:** 


### SURGERY.

ABLATION OF A GOITRE; CURE IN SIX DAYS.—Dr. A. Reverdin, Geneva. (*Journal de Médecine*, March, 1880, p. 119.)

Occasions for extirpating goitres are rarely met with. Still, this difficult and dangerous operation has its partisans nowadays. It still remains a difficult operation, but its dangers are very greatly diminished by the rapidity of the phenomena of reparation by the present methods. On this account our readers will be interested by the following case of Dr. Reverdin, of Geneva.

Mdlle. X., 33 years of age, of good constitution, presented herself to me for the cure of a goitre which she had carried for several years, and which had resisted all medical treatment. I found in the thyroid region an irregular tumor, very hard in certain points, soft and elastic in others ; it was more developed to the right than to the left, and had notably displaced the larynx and trachea toward the right side. Its volume was that of a medium-sized apple.

The patient asked if an operation was possible. Possible, yes ; but not absolutely indicated, if the danger attendant upon it was considered. The patient then, without hesitation, asked that the operation should be tried, and fixed the day herself. The operation was performed December 13. It had been agreed upon with the patient that chloroform should not be used. Everything was prepared to use Lister's method in the strictest manner.

The patient lay on her back ; a round pillow placed under the neck ; then, having rendered the part insensible with the ether spray an incision ($4\frac{1}{2}$ inches) 12 centimeters in length was made in the median line ; with grooved director and bistoury, the tumor was gradually approached ; arriving upon it, the difficulties commenced, large vessels, arteries and veins forming a veritable network in which one could only advance very slowly and prudently. Ligatures were difficult ; the number of clamps used (25) twice proved insufficient, and vessels had to be tied to liberate them, and allow the operation to continue. The efforts were directed to the right ; the finger passed toward the inferior part of the tumor was unable to lift it up. It was finally accomplished with great difficulty, and by the use of many ligatures. The second part of the operation was facilitated by the laxness of the tissue situated under the tumor ; however, towards the upper part, many more ligatures had to be employed. More than eighty ligatures were used, the catgut being excellent, and never breaking. The tumor was entirely extirpated. After a careful cleansing, and repeated with the strong solution, a caoutchouc drainage tube, the size of the finger, and ($2\frac{1}{2}$ inches) 7 or 8 centimeters long, was placed in lower angle of the wound, that is, behind the sternal notch ; ten sutures of catgut closed exactly the rest of the wound. A large compress covered the neck as far back as the ears, and to the shoulders and chest. Muslin bandages kept it in place, and formed a sort of cuirass, preventing any movement. The operation, including the dressing, lasted two hours and a half. The patient had admirable patience and courage, which singularly facilitated the surgeon's task. A little difficulty in swallowing, and a little smarting of the wound, were the only pains of which the patient complained the first two days. The dressing was renewed the day after; the wound looked well. The third day the drainage tube was removed; a large blood-clot entirely filled the opening, and was left undisturbed; no injection was made, the dressing simply being renewed. The patient swallowed better, especially food somewhat solid. The sixth day the dressing was removed, reunion being perfect; there was absolutely no discharge from within; the clot was organized, and a large granulation alone marked the place occupied by the drainage tube for three days. Some strips of diachylon were placed for safety over the region.

In six days, then, this cavity, in which remained a considerable number of ligatures, with a canal kept open for three days by a large trainage tube, was absolutely obliterated. There was never the slightest swelling; never pain, except slight difficulty in deglutition; a temperature and pulse nearly always normal, the highest point being 38.3° C. (100.9° F.), pulse 96, the second day, in the evening; save on this day, the temperature was never up to 38° C. (100.4° F.).

As a final result, there remains only a small, red, thread-like line, with dots to right and left at the point of sutures. The author has no comments to make upon the case, except to say that it was difficult, and in spite of excellent assistance, lasted two hours and a half, and that without Lister's method a similar result could not have been hoped for.

THE PORRO OPERATION.—(*Bulletin de l'Académie de Médecine*, No. 10, 1880.)

M. J. Lucas-Championnière presented two women to the Academy who had had successful Porro operations performed upon them. M. Lucas-Championnière has operated four times, with successful results to the mother in two, and four living children. These four cases of extremely narrow pelvis have presented themselves within two months. All the women had rachitic pelves, with a conjugate diameter of (about $2\frac{1}{3}$ inches) about 6 centimeters or less.

The first, Eliza A., aged twenty-seven, primipara, entered the Maternity on October 27, 1879, presenting a type of rachitism; her height was (49 inches) 1.25 meters. A first measurement of the pelvis gave a sacro-sub-pubic diameter of (3 inches) 0.078 m. A second measurement gave 0.073 m. M. Lucas-Championnière kept the woman under surveillance at the maternity during the last three weeks of her pregnancy; he had her examined by M. Tarnier, who approved of his project of intervention, being very much in favor of the Porro operation. On the morning of the 19th, labor began, without rupture of the bag of waters. Slight sanguineous discharge; disappearance of the neck. The operation was decided upon for the afternoon. At half-past three, all the precautions being taken, the operation was commenced. A large incision in the median line of (about 6 inches) 15 or 16 centimeters, passing a little above the umbilicus. On incision of the uterus, a frightful flow of blood occurred. The section finished, a living female, weighing (5 lbs.) 2 kil. 700 grams, was extracted by the feet, then the placenta, and with forceps the uterus was drawn forwards and two pins passed through the inferior segment; beneath, an iron wire; between the two, a second wire, which were tightened with Cintral's ligator. The uterus and ovaries were then removed. Six deep and one superficial sutures of the abdominal walls; per-chloride of iron applied to the stump, and complete Lister dressing. Duration of operation, three-quarters of an hour. The temperature never rose above 38.9° C.—that was the evening of the 21st. The sutures were removed successively until the 28th, and then the pins. The pedicle came away the thirteenth day. Dec. 20th, complete cicatrization. About six weeks after the operation, the patient got up completely cured. She presents to-day a perfect state of the abdomen, with a slight excavation. The touch reveals a large and mobile cervix. She has even had sexual connection already, without accident, the 10th of February. The child of this woman having been taken to the church for baptism the third day, after having been very lively, was chilled in the severe cold, became sick and died in thirteen days.

The second patient, Adele I., entered the Necker hospital on December 30. She was twenty-three years of age, and also rachitic; height, (50 inches) 1.30 m.; sacro-sub-pubic diameter, 0.067 m.—that is, a sacro pubic diameter of (2 inches) 5 centimeters. She had been in labor thirty-six hours, and the waters had been escaping for twenty-four hours. M. Lucas-Championnière operated December 30th, at 9 p. m., after having given chloroform for several hours, to calm an insupportable agitation. At this time the patient was in full labor, with a dilatation the size of the palm of the hand. An incision 16 centimeters long was made in the abdominal wall, commencing just above the umbilicus, and descending not quite so low as in the preceding case. The uterus was opened with considerable hæmorrhage. The child was extracted, breathed well, and weighed more than ($6\frac{3}{5}$ lbs.) 3 kilograms. Pins, wires and deep sutures as before; pedicle placed in lower angle of wound, much higher up than in the former case; Lister's dressing. No accident followed, save an extraordinary acceleration of the respirations, 55 in the minute, at the end of twenty-four hours. She never attained so much as 38° C. (100° F.). Dressing changed first the fifth day, then on the ninth day. At the second dressing the pedicle had fallen; at the ninth dressing, at the end of January, there remained but a very superficial ulceration. She began to get up in February, and for a long time has been in good health. The child, confided to a nurse, is doing finely. In this woman the union is so perfect that one can see but few traces of the operation, though it is so recent.

Another woman was operated upon the 3d of December; she died on the 5th, thirty-six hours after the operation. The child lives.

A fourth case at the Cochin hospital was operated on the 17th of January, 1880. Sacro-pubic diameter of (1.9 inches) 49 millimeters. Very well at first, she had a violent nervous attack after four hours, and died at the end of twenty-three hours. The child lived three days.

The author observed that all those cases of pelvis with a sacro-pubic diameter of 6 centimeters or less, only comprise women exposed to an enormous mortality under cephalotripsy. Cases which should not be confounded with those where the antero-posterior diameter approaches (2¾ inches) 7 centimeters.

M. Lucas-Championnière does not yet regard the Porro operation, as some have done, as worthy of replacing in all cases the Cæsarean operation.

The cause of death, as in hysterotomy, is inherent to resection of the uterus and its constriction, and the accidents observed are probably of a reflex order, due to shock to the nerves of the broad ligament. Consequently, except great modifications are made in the process, the operation remains one of menacing gravity. Up to the present time, no indications as to the mode of operating have been given. The author believes it easy to demonstrate, according to his own observations, that the abdominal incision should be made much higher than heretofore; one is much more sure of being able to make the dressing aseptic, in getting away from the pubis. This he obtained perfectly in the second case, and nearly so in the first one. The uterus is always incised too low. It would very probably be less grave if only a portion of moderate extent were cut away. The most perfect antiseptic precautions should be taken. It is probably more advantageous to the woman to interfere before labor. With antiseptic precautions, a specially clean locality is not necessary, as shown by the cure of his last patient in a badly-aired room, which had even served to isolate patients with contagious disorders. The operation requires isolation, warmth, repose, good care, incessant antiseptic precautions, and sufficient comforts; also skilled assistants.

